# Exploring the Gas Permeability of Type IV Hydrogen Storage Cylinder Liners: Research and Applications

**DOI:** 10.3390/ma18133127

**Published:** 2025-07-01

**Authors:** Xinshu Li, Qing Wang, Shuang Wu, Dongyang Wu, Chunlei Wu, Da Cui, Jingru Bai

**Affiliations:** 1 Engineering Research Centre of Oil Shale Comprehensive Utilization, Ministry of Education, Northeast Electric Power University, Jilin 132012, China; lixinshu@jlict.edu.cn (X.L.);; 2 School of Mechanical and Electrical Engineering, Jilin Institute of Chemical Technology, Jilin 132022, China; 3 Department of Chemical and Materials Engineering, University of Alberta, Edmonton, AB T6G 1H9, Canada; 4 Carbon Neutral Institute, Tsinghua University, Beijing 100084, China

**Keywords:** polymer materials, hydrogen storage, gas tank, liner, permeability

## Abstract

As hydrogen fuel cell vehicles gain momentum as crucial zero-emission transportation solutions, the urgency to address hydrogen permeability through the polymer liner becomes paramount for ensuring the safety, efficiency, and longevity of Type IV hydrogen storage tanks. This paper synthesizes existing research findings, analyzes the influence of different materials and structures on gas permeability, elucidates the dissolution and diffusion mechanisms of hydrogen in plastic liners, and discusses their engineering applications. We focus on measurement methods, influencing factors, and improvement strategies for liner gas permeability. Additionally, we explore the prospects of Type IV hydrogen storage tanks in fields such as automotive, aerospace, and energy storage industries. Through this comprehensive review of liner gas permeability, critical insights are provided to guide the development of efficient and safe hydrogen storage and transportation systems. These insights are vital for advancing the widespread application of hydrogen energy technology and fostering sustainable energy development, significantly contributing to efforts aimed at enhancing the performance and safety of Type IV hydrogen storage tanks.

## 1. Introduction

With the ongoing global attention to carbon emissions [1,2,3,4,5], hydrogen energy has emerged as a hot research topic at the current stage due to its zero-carbon, high-calorific-value clean energy [6,7,8]. The diversity of hydrogen energy sources and its wide range of applications make it a crucial direction for global energy upgrade and transformation [9,10,11,12]. The global prominence of hydrogen energy is not only evident in government support and policy backing but also in the active involvement and technological innovation within the industry [13,14,15]. Countries around the world have been actively formulating strategic plans and policy support measures for hydrogen energy development, increasing investment in hydrogen technology research and industrialization [16,17,18,19,20]. Simultaneously, an increasing number of enterprises and research institutions are actively exploring and innovating in the field of hydrogen energy, driving forward the advancement and application of hydrogen technology [21].

As technology matures and markets develop, hydrogen energy is gradually moving towards commercialization and industrialization [22,23], gradually being applied in multiple sectors such as transportation [24,25], energy storage [26,27,28], and industrial production [29]. In the future, hydrogen energy is expected to become one of the important solutions to global challenges such as energy security, environmental protection, and sustainable development [30,31]. Therefore, the emergence of hydrogen energy as a global hotspot has become an inevitable trend, with its development prospects highly anticipated.

Type IV hydrogen storage cylinders are high-pressure containers used for storing hydrogen gas. The internal structure and manufacturing materials of the cylinders must be capable of storing as much hydrogen gas as possible while ensuring safety. Their high strength and pressure-resistance performance ensure the safe storage of hydrogen gas [32,33]. With the increasing awareness of environmental protection, more and more consumers are inclined to choose clean energy vehicles to reduce the harm caused by carbon emissions to the environment. Hydrogen fuel cell vehicles, as zero-emission transportation, are rapidly seizing the market, and hydrogen storage cylinders, as the energy supply equipment for vehicles, provide effective guarantees for the market expansion of hydrogen fuel cell vehicles [34,35]. At the same time, the development of Type IV hydrogen storage cylinders contributes to the transformation of the energy structure, reducing dependence on traditional fossil fuels and promoting the development and utilization of clean energy.

Currently, Type IV hydrogen storage cylinders have excellent prospects for promotion. Hydrogen is an abundant energy resource that can be produced in various ways, such as water electrolysis, solar photovoltaic electrolysis, and biomass gasification, with high renewability [36,37,38]. Hydrogen storage cylinders use hydrogen as fuel, and the only by-product produced during combustion is water. They do not emit greenhouse gases such as carbon dioxide or pollutants, thus helping to reduce environmental pollution and address climate change issues [31,39,40,41]. Type IV hydrogen storage cylinders can be used in various fields such as fuel cell vehicles, energy storage systems, and industrial production, offering broad application prospects and market demand.

Currently, there have been no reported reviews on the research and applications of Type IV hydrogen storage cylinder liners’ permeability. Additionally, there is a lack of in-depth exploration of the factors influencing the permeability of plastic liners and of a comprehensive summary of research methods within the research field. This review meticulously summarizes the following aspects:

(1) Regarding the gas permeability of plastic liners in Type IV hydrogen storage cylinders, this review provides a detailed introduction to the working conditions of plastic liners and compares their advantages with other forms of hydrogen storage. It clearly discusses and explores the key considerations in the selection of liner materials, focusing on compatibility with hydrogen, aging resistance, and mechanical properties.
(2) Reveals the factors influencing the gas permeability of Type IV hydrogen storage cylinders, introducing the effects of material properties, temperature, pressure, and cylinder winding layers on gas permeability.
(3) Clarifies the mechanism of hydrogen molecule dissolution and diffusion in polymer materials, compares and analyzes international standards for hydrogen permeation testing, and introduces the application of numerical simulation methods in hydrogen permeation through polymer materials.
(4) Elucidates the safety of hydrogen storage systems, reviews the application of liner materials and manufacturing methods, and provides a comprehensive summary and discussion.

## 2. Permeability of Gas in Type IV Hydrogen Storage Cylinder Liners

### 2.1. Overview of Hydrogen Storage Cylinder Liners

The inner liner of a Type IV hydrogen storage cylinder is a crucial component responsible for storing, protecting, and isolating hydrogen gas. Situated inside the hydrogen storage container, it plays a pivotal role in hydrogen storage. Designing and manufacturing the liner requires high technical precision. A high-performance liner must first possess excellent gas tightness to prevent hydrogen gas from leaking or seeping out of the cylinder, ensuring the stability and safety of hydrogen storage. High-pressure hydrogen gas poses significant risks [42], requiring the inner liner to have sufficient strength and pressure resistance to prevent gas leakage and container explosions, among other safety incidents [43]. Compared to Type III cylinders with a working pressure of 35 MPa, Type IV cylinders can withstand a working pressure of up to 70 MPa, allowing them to store more hydrogen. However, this also increases the manufacturing difficulty and risks associated with the cylinders. Although accidents resulting from hydrogen storage container explosions are not common, they still occur, with the majority of incidents caused by hydrogen leakage leading to explosions [44,45,46,47].

The quality of onboard hydrogen storage cylinders significantly impacts the driving range of hydrogen fuel cell vehicles. Lightweight Type IV hydrogen storage cylinder liners, with their superior performance, can greatly increase the range of hydrogen fuel cell vehicles [35,48,49,50]. Therefore, while ensuring the service life, the thickness of the liner walls should be minimized as much as possible to reduce the weight and load of the entire hydrogen storage system. However, achieving this goal still presents a challenge given the current technological level [51].

The design and material selection of the inner liner need to consider the long-term stability and durability during extended use, ensuring that the performance and safety of hydrogen storage cylinders remain stable over time. The standard value for the hydrogen permeation rate in hydrogen storage cylinders is 6 NmL/(h·L) [52]. Hydrogen, being the smallest atom on the periodic table, possesses strong penetrative properties through plastic liners. During the hydrogen filling process, the Joule–Thomson effect [53,54] causes an increase in temperature as hydrogen passes through the valve and undergoes throttling. As the gas is pressurized inside the cylinder, the temperature further rises. Therefore, the inner liner of hydrogen storage cylinders needs to have a certain degree of heat resistance and stability [55] to prevent increased permeation due to temperature, which can lead to instability and bubbling or cracking of the plastic liner [56].

### 2.2. Liner Material Selection and Characterization

The liners of Type IV cylinders are typically composed of polymer materials. Internationally renowned manufacturing companies often use nylon (PA) and high-density polyethylene (HDPE) as raw materials for processing Type IV cylinder liners. Toyota, a Japanese company [57,58,59,60], used PA6 as the liner material for the onboard hydrogen storage cylinders in their early Mirai fuel cell vehicles. Products manufactured by Quantum Fuel Systems LLC and Hexagon Lincoln [61] utilize HDPE as the material for the liners of Type IV cylinders. A South Korean cylinder manufacturing company uses a nanocomposite material prepared from polyethylene and clay for processing the liners [61]. In comparison to the research history and practical market use of Type IV cylinders by foreign cylinder manufacturers, research on Type IV cylinder liners in domestic contexts in China is currently receiving considerable attention. It is anticipated that in the future, China will join the intense international competition in this field.

#### 2.2.1. The Compatibility of Materials with Hydrogen Gas

**Figure 1:**
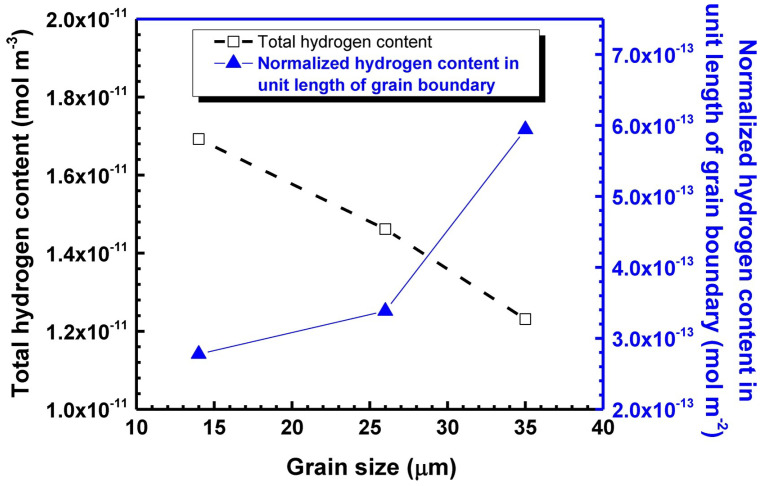
Total content of diffusible hydrogen and normalized hydrogen content in unit length of grain boundary with respect to grain size [72]. Copyright 2017, Elsevier.

In addition to traditional materials like PA and HDPE, more novel nanocomposite materials are being utilized in the manufacturing of Type IV hydrogen storage cylinder liners [62,63]. During the selection process of liner materials, compatibility with hydrogen gas must be prioritized [64]. This is because there are compatibility differences between polymers and metals in a hydrogen environment [65,66]. Polymer materials are sensitive to temperature, so it is essential to consider the effects of temperature and testing rate on polymers during testing and preparation processes. For polymers, their properties result from various factors, such as chemical structure, molecular weight, molecular chain distribution, and processing techniques. Different processing methods applied to the same polymer can yield different material properties. Processing methods like extrusion molding and injection molding directly influence the molecular chain orientation of polymers. Additionally, the crystallinity of polymers varies due to different cooling rates during the melting process. In the process of polymer modification, coupling agents are often used as additives to ensure uniform mixing with fillers and to reduce phenomena such as molecular aggregation. Hydrogen atoms have a small atomic radius, allowing hydrogen to enter metals in atomic form. For hydrogen-filled storage cylinders, metal liners mainly endure the hydrogen embrittlement caused by high-pressure hydrogen dissolution [67,68,69]. However, the occurrence of hydrogen embrittlement also depends on the degree of grain refinement of the material as well as the effect of inclusions; fine grains contribute to the detachment of hydrogen from normal lattice sites, dislocations, and grain boundaries, and an increase in the grain boundary area drives a decrease in the amount of normalized hydrogen captured per unit length of the grain boundary (Figure 1) [70,71,72]. Polymers, on the other hand, do not experience hydrogen embrittlement. This is because hydrogen enters polymers in molecular form. Therefore, materials like polyethylene, polypropylene, and nylon have excellent compatibility with hydrogen gas, making them suitable for processing into plastic liners.

#### 2.2.2. The Aging Resistance of Materials

Due to the influence of external environmental factors such as temperature and humidity during the operation of fuel cell vehicles [73,74], these fluctuations can lead to degradation and discoloration of the polymer materials used in Type IV hydrogen storage cylinders [75], affecting the cylinders’ lifespan [76]. Elevated temperatures promote the oxidation of polymers, causing autonomous fracture of the polymer main chains in the presence of oxygen [77]. This dual action of heat and oxygen, known as thermal oxidation, is the primary cause of aging in polymer materials. Polymers containing amide groups (-CONH-) are particularly sensitive to thermal oxidation [78].

**Figure 2:**
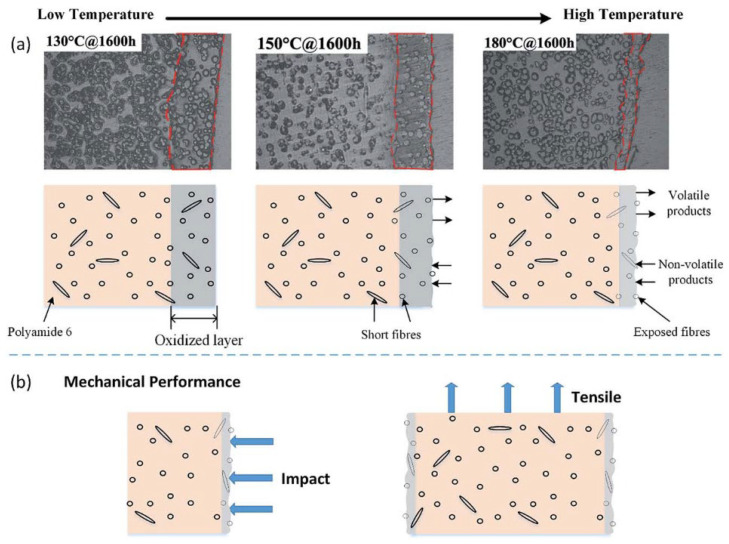
Schematic model of mechanical performance of short fiber-reinforced polyamide 6 composites for thermo-oxidation ageing for different ageing temperatures. (**a**) Thermal oxidation aging results (**b**) Mechanical performance [83]. Copyright 2017, RSC.

**Figure 3:**
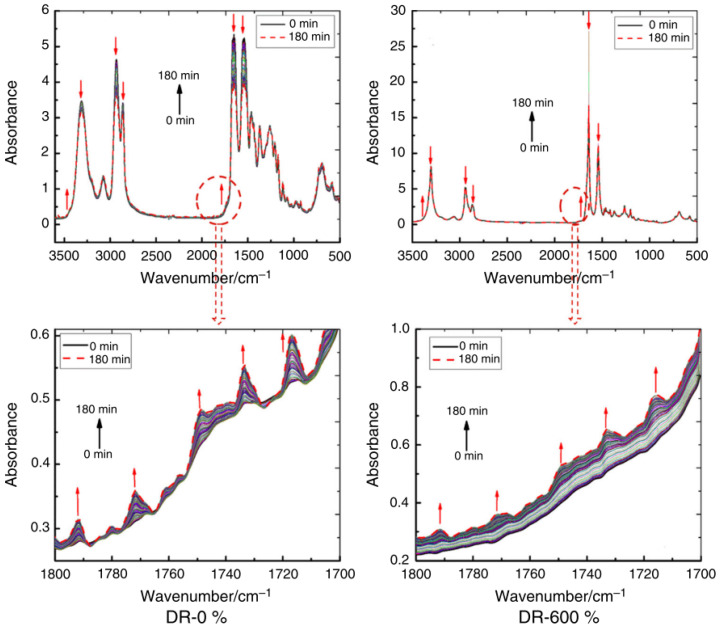
In situ FTIR spectra of PA6 with different draw ratios [84]. Copyright 2016, Springer Nature.

Researchers assess the hydrogen barrier performance of liners by measuring the hydrogen permeation rate of HDPE through thermal cycling and exposure to high-pressure hydrogen. Additionally, they fit the gas permeation rate of cylinders during their lifespan, contributing significantly to the advancement of manufacturing standards for hydrogen fuel cell vehicles [79]. Alexis et al. [80] investigated the effects of thermal oxidation on the tensile and fatigue properties of thermoplastic plastics. They found that when the fiber content increased from 35% wt. to 50% wt., the overall impact of aging on the material decreased. They also established a database suitable for analyzing aged materials, providing a foundation for studying the aging lifespan of thermoplastic materials. Gijsman and colleagues [81] utilized luminescence measurement techniques to ascertain the thermal initiation correlation of oxidative reactions in aliphatic compounds, semi-aromatic polyamides, and polyesters. Research shows that the intensity at 200 °C is 100 times that at 140 °C. They compared the thermal initiation correlation of polyethylene UHMwPE (Ultra-High Molecular Weight Polyethylene), and the research findings diverged significantly from expectations. The formation of thermal alkyl radicals triggers oxidation, playing a crucial role in aliphatic polyamides. Schubert et al. [82], using characterization analysis methods, investigated the thermal aging of PA12 powder used for selective laser sintering and sintered specimens in nitrogen and air. The study found that temperature and aging time are critical factors influencing molecular chain degradation. Research by Sang et al. [83] discovered that during thermal oxidation, the surface oxidation layer formed on PA6 does not thicken with prolonged oxidation time (Figure 2), and the maximum thickness of the oxide layer reached 150 μm. Instead, the established oxidation layer acts as a barrier against oxygen, preventing further oxidation of the internal material. Shi et al. [84] determined that molecular orientation plays a dominant role in enhancing the thermal oxidation stability of PA6 by preparing highly oriented PA6 (Figure 3). Meanwhile, the highly oriented PA6 reduced the oxygen permeability coefficient by a factor of 1.3. In studies on measurement techniques for polymer oxidation rates, researchers have employed respirometers in specific experimental setups. This type of dual-channel respirometer, equipped with a fuel cell detector, exhibits high sensitivity and enables the precise short-term measurement of polymer oxidation rates. Consequently, it facilitates the prediction of polymer autoxidation at ambient temperatures [85]. Okamba-Diogo and colleagues [86] conducted thermal oxidation studies on PA11 films under various oxygen pressures and temperatures. The research revealed that the formation of hydroxyl groups is influenced by certain physical and macromolecular modifications. In subsequent comprehensive investigations, Okamba-Diogo identified chromatographic methods more suitable for analyzing PA11 [87] and examined the impact of categorized antioxidants on the thermal stability of PA11 [88], thereby enriching the research on the thermal oxidation aging of PA11. In earlier research by Forsström et al. [78], thermal oxidation studies were conducted on stable and unstable PA6 samples. The research indicated that while oxidation rate is influenced by peroxide concentration, other factors may play a more significant role. From a kinetic perspective, oxidation primarily targets amino groups, while C-H bonds are less susceptible. The instability of amino hydroperoxides associated with nitrogen-induced effects is also another factor contributing to PA oxidation [89,90,91].

#### 2.2.3. The Mechanical Properties of Materials

The operating pressure of Type IV hydrogen storage cylinders can reach 70 MPa, placing high demands on the mechanical properties of materials. Since the liner of Type IV cylinders is directly exposed to high-pressure hydrogen, research on the mechanical properties of plastic liner materials under hydrogen atmosphere is particularly important. However, researchers have found that, employing stress-strain testing of polymers under hydrogen atmospheres of different pressures below 10 MPa, neither the hydrogen atmosphere nor the exposure duration affects the tensile properties of the materials [92,93,94]. However, thermal oxidation has a more pronounced impact on the mechanical properties of polymer materials [78,80,83], as demonstrated in numerous research studies. Therefore, the mechanical properties of plastic liner materials primarily manifest in the formation of microcracks within the liner or brittle fracture due to impacts, significantly compromising the safety of the cylinder’s use. This could lead to hydrogen leakage, potentially causing explosion incidents and resulting in societal harm.

During the repeated filling and emptying processes in the use of Type IV hydrogen storage cylinders, it is essential to consider that the cylinder liner is wrapped with carbon fiber-reinforced composite material. The plastic liner not only bears the pressure from the hydrogen gas inside but also experiences pressure from the carbon fiber composite wrapping layer, making the load situation extremely complex. Additionally, hydrogen gas permeating from the liner gathers at the interface between the liner and the wrapping layer, gradually creating external pressure. During the rapid depressurization of the cylinder, the plastic liner is highly prone to instability, leading to radial buckling deformation and eventual collapse failure. The occurrence of this specific failure mode presents challenges in the design, manufacture, and material selection of cylinder liners. Research by Lin et al. [95] on the typical axial compression of liners has confirmed that minor geometric defects have a significant impact on liner collapse. With the widespread use and advancement of finite element software, an increasing number of researchers are opting to utilize finite element analysis to study the collapse instability of plastic liners [96]. Earlier studies were primarily focused on the initial stages of collapse damage caused by hydrogen permeation [97,98,99], lacking a certain level of completeness. However, recent studies have made significant progress in simulating the process of collapse damage, providing a comprehensive explanation for the collapse instability mechanism of Type IV hydrogen storage cylinders [100]. Nouri et al. [101] conducted a life calculation of Type IV CNG composite cylinders, conducting experimental analyses on the mechanical and fatigue properties of different liner materials. Additionally, they utilized finite element analysis for transient dynamic analysis, obtaining the temporal variation of stress tensors in critical regions. Based on existing standards, this life assessment is highly accurate, with an error of only 15% when compared with experimental data. Santharam et al. [102] established a fatigue database by conducting extensive tensile and compression tests on 50% glass fiber reinforced PA66, defining a new fatigue criterion that accurately describes the integrity of the database with fewer parameters. The addition of fiber-reinforced composite materials to polymers often has a significant effect on mechanical properties and service life [103,104,105,106,107,108]. Backens et al. [109] investigated the mechanical properties and permeability of cyclically aged HDPE, XPE-20, XPE-45, PA6, and PA12. The study showed that aging had a minimal impact on mechanical properties but significantly affected gas permeability. Under ageing conditions at 200 °C, cross-linking by peroxide can reduce permeability by approximately 50%.

As is well known, the commonly used matrix material PA6 for plastic liners has strong hygroscopicity, and this hydrophilic behavior leads to significant changes in the mechanical properties of the liner material with moisture content [110,111]. Therefore, in the preparation process of plastic liners, it is essential to strictly adhere to the processing requirements, ensure temperature and relative humidity control, and guarantee that the plastic liners have stable and excellent mechanical properties.

### 2.3. The Influencing Factors of Gas Permeability

For Type IV hydrogen storage tanks, the gas permeability of the plastic liner directly affects the performance of the tank. The polymer material of the plastic liner needs to have excellent gas barrier properties [112,113]. In recent years, some representative polymer materials with their gas barrier properties and mechanical performance are shown in Table 1. There are many internationally recognized methods for improving gas barrier properties that have garnered considerable attention. By improving the processing techniques to optimize the polymer structure, lamination [114,115] has been widely used as a processing technique in recent years. Similar methods include multilayer coextrusion technology [63] and twist extrusion lamination technology [62,115]. These optimized processing methods can enhance the orientation of polymer materials and reduce the free volume penetrated during gas permeation. Generally, improved processing techniques are more suitable for materials with relatively slow crystallization rates, as it is difficult to control the process when crystallization occurs too rapidly. Researchers have attempted to incorporate sheet-like materials into polymer materials to enhance the gas barrier properties of composite materials [62,116]. The potential barriers of polymers can be improved by increasing the intermolecular forces between polymer chains. Polar functional groups and planar ring structures in polymer molecules can easily form hydrogen bonds, which reduce the free volume of polymers and restrict gas molecules, thereby enhancing the gas barrier properties of the material [117]. Additionally, methods such as melt blending and forming a dense oxide layer on the surface of the material [118] can also improve the gas barrier properties of polymer materials.

#### 2.3.1. The Impact of Inner Liner Materials

Different polymer materials exhibit variations in gas permeability [129]. The gas barrier properties and mechanical performance of polymers commonly used in Type IV bottle liners, such as PA, are generally superior to HDPE, although HDPE is easier to process into shape [129,130]. In the selection of matrix materials, researchers tend to prefer materials with comprehensive performance advantages as the matrix. In an earlier study by Olivier et al. [120], the gas barrier properties of PA6 composite films were investigated. Researchers employed injection molding and blow molding techniques to incorporate unmodified α-ZrP inorganic nanofillers into the PA6 matrix. The study revealed that the properties of the crystalline phase and orientation play a crucial role in the gas barrier performance of the material. Lozay et al. [121] conducted a study on PE and PA6 films using a forced assembly coextrusion process with a layer multiplier element (LME) to prepare multilayer films with well-defined structures. The maximum number of layers can reach 2049, with a thickness of up to 50 nm. Although this film structure limited the improvement of the barrier properties, the change in crystal orientation played a role in enhancing the gas barrier performance of the material, while also increasing its stiffness and strength. Marais et al. [63], through similar research, found that only by filling the nano-fillers in the PA6 layer of PE/PA6 could the barrier properties of multilayer films be improved. Shi et al. [114] prepared film-like composite materials by laminating graphite and PE with different particle sizes using a laminating method. Although the study did not investigate the gas barrier properties of the composite material, it confirmed the excellent mechanical and dielectric performance through multiple characterization methods. Compared to a single-layer pure PE film, the 36-layer composite with a particle size of 15 μm exhibited improvements in tensile strength by 34.76% in the longitudinal direction and 68.39% in the transverse direction, while the dielectric constant doubled. These findings provide a solid foundation for the application of such materials in Type IV hydrogen storage cylinder liners. Liu et al. [119] mixed graphene (GF) with polyethylene (PE) and produced polyethylene/graphene composite materials with high gas barrier properties using a method called boundary-free unconstrained hot-pressing. This method induces stretching–shear coupling flow in the polymer melt, creating differences in shear flow between the melt surface and interior, which helps in the alignment of PE and GF molecules. The PE/GF composite materials prepared using this method showed a 98.6% improvement in hydrogen gas barrier compared to pure PE, with a 31.4% increase in tensile strength and a 21.1% increase in Young’s modulus.

Due to the low gas barrier properties of HDPE, research efforts aimed at enhancing its gas barrier performance often involve incorporating inorganic nanoplate materials into the HDPE matrix. Ma et al. [62] addressed the issue of low gas barrier properties in HDPE by incorporating montmorillonite (MMT) into the HDPE matrix to create nanocomposites. They also utilized the micro-nano torsional laminated extrusion (MNTLE) to enhance the uniform distribution of MMT in the HDPE matrix. This led to a 16.27% increase in crystallinity and resulted in improved mechanical strength. Specifically, the tensile strength increased by 46.18%, and the elongation at break increased by 27.37%. Remarkably, the gas barrier rate increased by 41.9%. Zhang et al. [123] utilized a multi-layer coextrusion technique to co-extrude HDPE with a novel cyclic olefin copolymer (COC) HP030, producing HDPE/HP030 multi-layer films. This technique constrained the spherical particles of HDPE material, effectively increasing the tortuosity of gas permeation through the material. When the thickness of the single-layer HDPE reached 290 nm, the material exhibited a doubling in oxygen barrier performance and an astonishing five-fold increase in water vapor barrier performance. Lei et al. [124] employed a micro-layer coextrusion technique to distribute HDPE and PA6 alternately. The researchers were surprised to find that this layered structure significantly enhanced the gas barrier properties. When the CP content reached 10 wt%, the nitrogen permeability coefficient decreased by approximately two orders of magnitude compared to the unmodified material. Meanwhile, Moghri et al. [116] investigated the barrier properties of HDPE/PA6/NC nanocomposite containers. They effectively evaluated the types and contents of compatibilizers using response surface methodology, demonstrating that the type of compatibilizer played a crucial role in the material’s barrier properties. Additionally, the team utilized MINITAB software (Minatab 17) to develop correlations, allowing for the prediction of material content correlations through software analysis, thereby saving on experimental costs.

Ethylene vinyl alcohol copolymer (EVOH) also exhibits excellent barrier properties against oxygen, carbon dioxide, and nitrogen [125]. This is attributed to the strong intermolecular bonds formed by the polar hydroxyl groups in the vinyl alcohol units. However, the presence of these polar hydroxyl groups imparts some hydrophilicity to EVOH [131,132,133]. Hassanpour et al. [66], by blending HDPE-g-mah with EVOH, introduced -COOH functional groups and proposed corresponding fracture mechanisms. They also demonstrated the significant role of compatibilizers in hindering crack propagation and interfacial adhesion. Souza et al. [126] studied the effects of the number of layers in EVOH/LDPE materials on oxygen permeation rates and thermal molding performance. Their research found that utilizing a continuous multi-layer coextrusion process to produce 32-layer EVOH/LDPE materials resulted in optimal gas barrier properties and tensile strength.

#### 2.3.2. The Influence of Temperature and Pressure

The temperature, pressure, and aging of the plastic liner in Type IV hydrogen storage cylinders are crucial factors influencing gas permeation [134,135], apart from inner lining materials and processing techniques. Increased pressure can prompt the plastic liner to reassemble tightly, shifting its relatively loose structure towards a denser configuration, thereby decreasing the permeation coefficient of hydrogen. However, in comparison to the rise in pressure, this reduction in permeation coefficient appears trivial [136].

**Figure 4:**
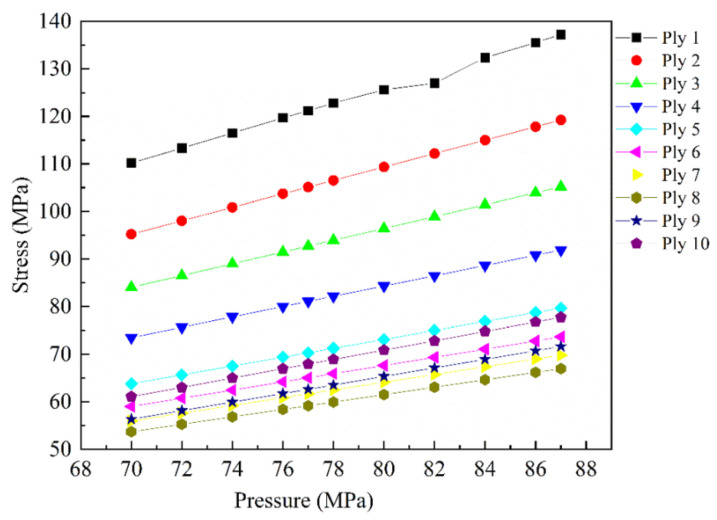
Maximum stress in plies of the composite structure at different pressure [96]. Copyright 2020, WILEY.

Temperature plays a crucial role in determining the solubility of hydrogen in the plastic liner material, consequently affecting its gas barrier properties. Moreover, temperature can impact the mechanical characteristics of the plastic liner. Lower temperatures may decrease the plastic liner’s toughness, making it susceptible to microcracks. When combined with low pressure, these cracks may propagate further, compromising the plastic liner’s ability to block gas and shortening the gas cylinder’s lifespan. Conversely, higher temperatures can directly increase the permeability of polymer materials to hydrogen and accelerate aging, ultimately reducing the gas cylinder’s lifespan [137,138]. For Type IV hydrogen storage cylinders, apart from environmental factors, the rapid flow of hydrogen inside the tank during the filling process leads to temperature elevation due to the conversion of kinetic energy into heat, the Joule–Thomson effect, and the rapid compression of hydrogen [96,139]. As temperature rises, polymer chains become more mobile, leading to an expansion in the material’s free volume. This, in turn, increases the potential for hydrogen leakage from the plastic liner. Moreover, elevated temperatures can trigger various aging reactions. Furthermore, as pressure increases, it leads to an increase in the maximum stress of composite materials (Figure 4). The accumulation of energy gradually induces chain breakage, alters the microstructure, and promotes the propagation of internal microcracks, ultimately resulting in stress fatigue failure. External impacts present significant challenges to the mechanical properties of liner materials, including strength, modulus, and elongation at break, which are further influenced by pressure, temperature, and duration of exposure [80,81,96,107,140].

During the depressurization process, the plastic liner of Type IV hydrogen storage tanks is highly susceptible to collapse. Pepin et al. [97] employed a combined approach of experiments and numerical simulations to investigate specific critical parameters such as pressure and temperature. Their study revealed that during depressurization, the pressure disparity between the interface of the plastic liner and the winding layer, as well as the pressure within the cavities on the inner surface of the plastic liner, are crucial factors contributing to liner collapse. Moreover, temperature significantly influences the yield point of the liner material, interface resistance, and hydrogen solubility. Hence, these factors may directly or indirectly lead to the collapse of the plastic liner. During the fluctuation of pressure levels, gases infiltrating the interior of plastic and rubber products can cause cavitation [141]. In the case of Type IV hydrogen storage tanks operating under high pressure conditions, the plastic liner is prone to foaming. Yersak et al. [142] conducted modeling of the foaming phenomenon in the plastic liner of Type IV hydrogen storage tanks [143]. Their research revealed that the foaming of the plastic liner is attributed to the infiltration of gas into the material under high-pressure conditions. While gases also permeate out of the material during this process, stress is induced when the depressurization rate of the tank exceeds the gas permeation rate, leading to the formation of bubbles in the liner material. This work lays the groundwork for predicting the selection and design of liner materials.

#### 2.3.3. The Impact of Winding Layers and Coatings

**Figure 5:**
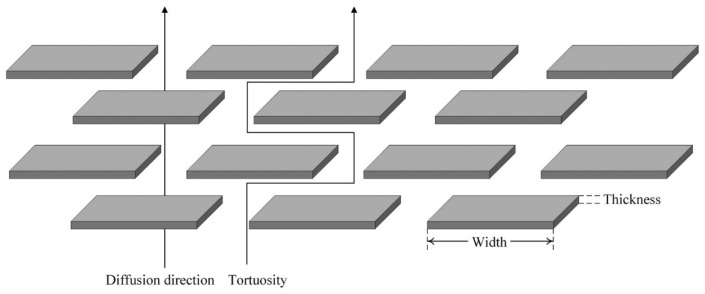
Increased tortuosity of diffusion paths of gas molecules [148]. Copyright 2023, MDPI.

Nuruddin et al. [144] conducted an in-depth study on the permeability of cellulose nanocrystals (CNC) and polyvinyl alcohol (PVA) coatings. Their research revealed that PVA materials enhance the diffusion rate of thin films by occupying the free volume of the coating material. While this study primarily focused on the field of food packaging, it also offers new insights for research into the gas barrier properties of Type IV hydrogen storage tank liners. Layek et al. [145] similarly explored the same matrix, employing a spray coating technique to produce layered GO/PVA nanocomposite films. This method of applying materials with high-gas-barrier properties onto the surface of the substrate demonstrated significant hydrogen gas barrier effects. Further research indicates that coating polymer surfaces with high-barrier materials can enhance the gas barrier properties of the materials. For instance, preparing multilayer films with chitosan, carrageenan, and montmorillonite coated on PET surfaces through solution coating forms a “brick-like” structure with staggered arrangement, increasing the tortuosity of gas molecule diffusion paths and enhancing the gas barrier properties of the material (Figure 5) [146,147,148]. Another approach to hydrogen storage involves storing high-pressure hydrogen gas at 70 MPa in microglass capillaries and capillary arrays, with temperatures maintained at 40–60 °C [149,150,151]. To ensure the mechanical performance of the storage device, polymers are used as coatings for protection. For instance, epoxy resin and glass paint play a facilitating role in the geometric optimization process of glass capillary storage devices [152]. This warrants consideration in the academic community because epoxy resin is used in the processing and manufacturing of the winding layer in Type IV hydrogen storage tanks. Therefore, further research is needed on the curing conditions and curing processes of epoxy resin regarding their effects on hydrogen permeation. Additionally, coating the inner and outer surfaces of the hydrogen storage tank’s plastic liner with coatings to further block hydrogen permeation is also worth contemplating.

Overall, the importance of gas permeability in Type IV hydrogen storage tank liners is self-evident, directly impacting the safety, efficiency, and reliability of hydrogen storage tanks. Research aimed at improving the gas barrier properties of plastic liners mostly involves modifications to polymer materials, such as adding layered fillers, or improving processing techniques. Fundamentally, these approaches aim to increase the density of functional groups within polymer molecules to enhance gas barrier properties. Regardless of the method used to modify liner materials, factors such as hydrogen compatibility, aging resistance, and mechanical performance during use cannot be overlooked. These are all potential areas of research for scholars studying Type IV hydrogen storage tank plastic liners. Moreover, besides temperature and pressure, there are numerous other potential factors influencing liner material gas permeability that warrant further exploration and discovery. Therefore, further exploration and innovation in this highly regarded topic are crucial for enhancing the gas barrier properties of Type IV hydrogen storage tank plastic liners.

### 2.4. Summary and Outlook of Gas Permeability in Liner Materials

As demonstrated throughout this section, the gas permeability of Type IV hydrogen storage cylinder liners is a key factor affecting their safety, durability, and energy efficiency. This chapter has comprehensively reviewed the selection of liner materials, their aging resistance, mechanical performance under hydrogen environments, and the influence of temperature, pressure, processing methods, and barrier coatings. Furthermore, several advanced material strategies, such as multilayer co-extrusion, nanofiller incorporation, and hybrid polymer systems, have shown promising potential in improving the gas barrier properties.

However, despite the progress made, current studies still face challenges in areas such as long-term stability under realistic operating conditions, interfacial behavior between liner and composite overwrap, and standardized permeability evaluation methods.

To clearly summarize the key findings, limitations, applications, and future prospects discussed in this chapter, Table 2 provides an integrated overview.

## 3. Study Methods for Gas Permeability of Liners

**Figure 6:**
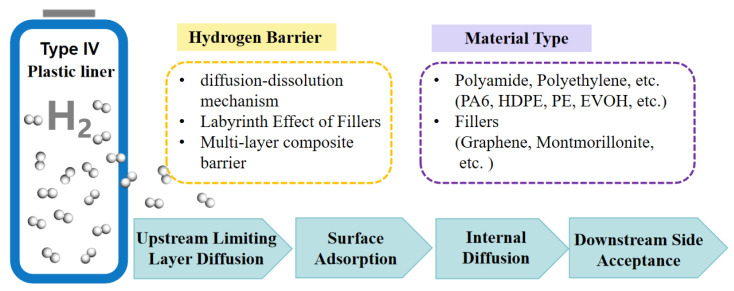
Schematic of gas diffusion in polymers.

The plastic liner of Type IV hydrogen storage cylinders does not experience hydrogen embrittlement like metallic materials do. However, hydrogen molecules can slowly permeate through the molecular structure’s free volume regions of thermoplastic materials. This process of gas molecule transport within polymer molecules is known as the dissolution diffusion mechanism [153]. The diffusion of gas within polymers involves multiple stages, as illustrated in Figure 6. Firstly, gas molecules need to pass through the upstream limiting layer on the surface of the polymer. This layer is typically composed of molecules near the polymer surface, which may interact physically or chemically with the gas molecules, leading to a slowdown in the diffusion rate of the gas molecules. Subsequently, the gas molecules enter the second stage, where they adsorb onto the polymer surface. This may be due to factors such as the chemical affinity or solubility of the surface. Gas molecules in this stage are adsorbed onto the polymer surface, thereby slowing down their further diffusion rate. Once the gas molecules overcome the resistance of surface adsorption, they enter the interior of the polymer. At this stage, gas molecules diffuse through the pores or intermolecular spaces of the polymer. This process is typically described by Fick’s law, where the diffusion rate depends on the concentration gradient and the diffusion coefficient within the polymer. Lastly, the gas molecules need to pass through another limiting layer similar to the first stage. This may be due to the presence of contaminants on the polymer surface or the influence of surrounding environmental conditions. The second to fourth stages of these diffusion steps play a decisive role [32,61,154].

Fick’s law is a fundamental principle that describes the diffusion of substances, including the relationship between diffusion flux and concentration gradient. For the diffusion of gases in polymers, Fick’s law [142] is expressed by the following Equation (1):

(1) $$J=-D\frac{\partial c}{\partial x}$$

In the equation, *J* represents the diffusion flux of the gas (mol/m^2^·s); *D* represents the diffusion coefficient (m^2^/s); *c* represents the molar concentration of the gas (mol/m^3^); *x* represents the direction of diffusion.

The dissolution of hydrogen in polymers follows Henry’s law [142], expressed by Equation (2):

(2) $$c=SP_{H}$$

In the equation, *S* represents solubility (mol/m^3^·Pa), and *P_H_* represents the partial pressure of the gas (Pa).

The permeability coefficient (*P*) is the product of the diffusion coefficient and the solubility coefficient [155], as shown in Equation (3):

(3) P=D×S

According to relevant international standards [64,156], ensuring the safety and reliability of the plastic liner in Type IV hydrogen storage tanks requires hydrogen permeation testing of the liner material. Experimental testing methods are widely employed for this purpose, as they allow for a more intuitive comparison of measurement results, facilitating data analysis and discussion [148,155]. With the increasing popularity of numerical analysis software, more and more scholars are turning to the numerical analysis of liner material permeability. By comparing with experimental data, they have also obtained reliable results [135,157]. Moreover, exploring new approaches for studying the permeability of Type IV hydrogen storage vessel liners has opened up clearer insights into the mechanism of hydrogen permeation in liner materials.

### 3.1. Experimental Measurement Methods

Due to the properties of hydrogen, its explosion limits are theoretically calculated to be 4.0% to 75.6% (volume concentration). To ensure the safety of onboard Type IV hydrogen storage tanks, it is essential to strictly control the hydrogen permeation rate of the tanks. According to ISO standards [158], the hydrogen permeation rate of Type IV hydrogen storage tanks should be less than 46 NmL/(h·L) at 1.15 times the nominal working pressure and a temperature of 55 °C. At 15 °C, the hydrogen permeation rate should be less than 6 NmL/(h·L) [158].

According to the safety standards for the use of Type IV hydrogen storage tanks, Canada and other countries have issued several related hydrogen permeation testing standards. Among the numerous testing standards, hydrogen permeation testing methods all require completion under a high-pressure hydrogen atmosphere, a method known as High-Pressure Hydrogen Permeation (HPHP) testing. The HPHP method requires a pressure differential between the test chambers, consisting of a high-pressure chamber and a low-pressure chamber. The sample is placed between these two chambers. When the hydrogen pressure in the high-pressure chamber stabilizes to meet the requirements, the amount of hydrogen permeating through the sample over time is recorded comprehensively. The extent of gas permeation between the two chambers determines the gas permeation coefficient. Fujiwara et al. [159] employed this technique to evaluate the permeability of five polyethylene materials under high-pressure hydrogen atmosphere, demonstrating that the test results obtained through the HPHP method remained reliable, even under high-pressure conditions.

**Figure 7:**
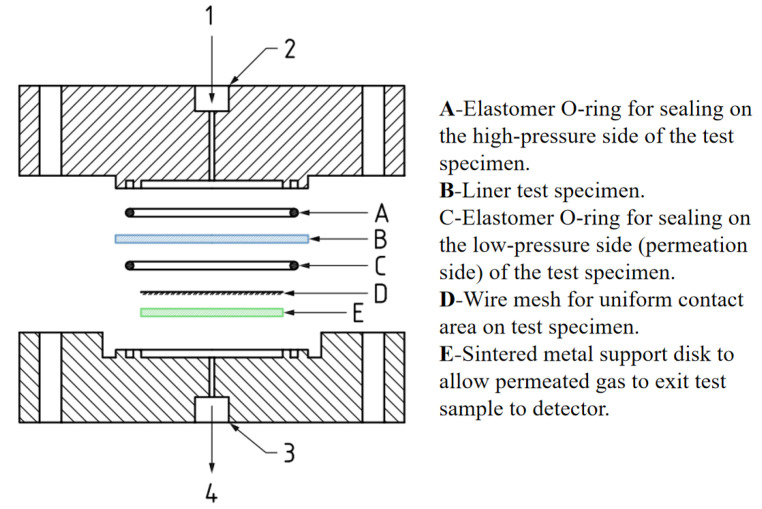
Expanded schematic representation of the high-pressure permeation cell. 1–High pressure gas, 2–High pressure gas port, 3–Low pressure gas port, 4–To detector [148]. Copyright 2023, MDPI.

Currently, the most widely used standards internationally comprise two: one is the CSA/ANSI CHMC 2 revised by the Canadian Standards Association and CSA America in 2019 [160], and the other is ISO 11114-5 [158] revised by the International Organization for Standardization in 2022 [158]. Both standards specify the HPHP method for testing, and the structure of the hydrogen permeation apparatus is also nearly identical, as shown in Figure 7. In 2020, China released the T/CATSI 02 007 standard [161], which is a group standard revised by the China Technical Supervision Information Association [148]. In 2023, led by Zhejiang University, the national standard GB/T 42610-2023 “Test Methods for Plastic Liners and Hydrogen Compatibility of High-pressure Hydrogen Cylinders” was published [122], and this standard was formally implemented in June 2024. It is believed that the formal implementation of this standard will contribute to more remarkable achievements in the research of hydrogen permeation in Type IV hydrogen storage cylinder liners.

**Figure 8:**
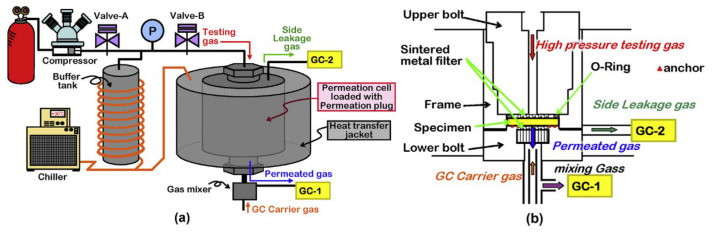
(**a**) Schematic of permeation system. (**b**) Schematic of permeation plug [127]. Copyright 2020, Elsevier.

The measurement methods for the hydrogen permeation coefficient are not limited to the HPHP method; the Thermal Desorption Analysis (TDA) method is also used to test the hydrogen permeation coefficient. The TDA method is a non-steady-state test capable of rapidly assessing the total dissolved hydrogen, but it is prone to distortion during the decompression process. In contrast, the HPHP method is based on steady-state flow measurements, requiring longer testing time while delivering higher precision in results. Fujiwar et al. [127] compared the test values from their developed device (Figure 8) with those obtained via the TDA method, indirectly demonstrating the reliability of TDA. However, compared to TDA, the HPHP method provides more dependable results when measuring hydrogen permeability under high-pressure equilibrium conditions [127,159]. In recent years, the pressure difference method has been primarily used for gas permeability testing apparatus, especially suitable for membrane materials [155]. This includes methods such as time lag measurement, flow rate measurement, or curve fitting.

Different standards have varying requirements for measurement conditions such as temperature and pressure. According to the permissible temperature range of hydrogen storage cylinders during use, which is −40 to 85 °C, the allowable upper temperature limit is 85 °C. However, in normal usage scenarios, it is rare for ambient temperatures to reach 85 °C, even in regions with consistently high temperatures where surface temperatures may exceed 50 °C. Vehicles operate outdoors while driving and during refueling processes, so even if hydrogen permeates into the external environment, it is unlikely to accumulate to high concentrations, thereby minimizing the risk of accidents. However, if vehicles are parked in poorly ventilated spaces such as garages for extended periods, the situation could be vastly different. Relatively confined spaces provide favorable conditions for hydrogen permeating into the air. Once explosive conditions are reached, safety incidents can occur easily, resulting in incalculable losses. Therefore, conducting permeation tests under the conditions of 55 °C and 15 °C nominal working pressure, as required by standards, is reasonably justified [158,160]. However, current standards do not address hydrogen permeation testing under the low temperature conditions of −40 °C. Low temperatures can cause a decrease in the mechanical properties of materials, and over prolonged usage, they can lead to the extension and expansion of microcracks in the inner lining material. These factors can significantly increase the hydrogen permeation rate of the plastic inner liner of Type IV hydrogen storage cylinders [162,163], thereby potentially triggering safety incidents.

When testing plastic inner liner samples, it is important to ensure that environmental factors such as humidity and temperature do not interfere with the measurements, as they can cause deviations in the results. However, it is also crucial to consider the working environment of vehicle-mounted hydrogen storage cylinders. If necessary, an analysis of the permeability of the plastic inner liner should be conducted with targeted measures against high temperature and humidity factors [124,164].

### 3.2. Numerical Simulation Methods

While experimental methods provide more intuitive measurement results, they are limited by the resolution of the technology as well as by constraints in time and spatial scales. Molecular dynamics simulation methods play a crucial role in studying the microstructure and dynamic properties of polymer molecules, offering a way to overcome the limitations and shortcomings of experimental methods [165,166].

**Figure 9:**
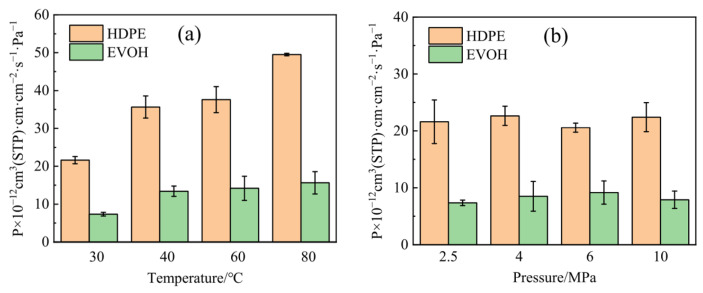
Permeability coefficient of hydrogen in HDPE and EVOH: (**a**) 2.5 MPa; (**b**) 30 °C [170]. Copyright 2024, MDPI.

Currently, researchers have conducted molecular dynamics simulations on the dissolution and diffusion of molecules in polymers, which include not only gas molecules but also liquid molecules. Karlsson et al. [167] employed molecular dynamics to investigate the diffusion of oxygen in polyvinyl alcohol. By varying the water concentration in polyvinyl alcohol, they discovered that water molecules disperse uniformly within the polymer. Pavel et al. [168] employed molecular dynamics simulations to investigate the diffusion behavior of oxygen and carbon dioxide in amorphous polyethylene terephthalate glycol at different temperatures. The simulated gas diffusion coefficients indicated an exponential variation with increasing free volume. Cha et al. [169] utilized quantum and molecular dynamics simulations to study the molecular behavior of hydrogen in polymer proton exchange membranes, deeply exploring the permeability of hydrogen into the membrane. They ultimately determined that the hindrance effect of long side chains impedes hydrogen permeation. Zhang et al. [170], focusing on two polymers, HDPE and EVOH, researched the permeation characteristics and mechanisms of hydrogen gas. Their study revealed that both HDPE and EVOH polymers exhibit high sensitivity to temperature but weak sensitivity to pressure, as shown in Figure 9. Additionally, hydrogen interacts more strongly with HDPE, making it more prone to hydrogen adsorption, thus elucidating the microstructural advantages of EVOH materials in terms of hydrogen barrier properties. Voyiatzis et al. [171], through their study on the solubility of hydrogen in PA6 and HDPE polymers, found that hydrogen and oxygen molecules cannot penetrate the polymer crystals, and gas migration mainly occurs in the free volume. They used molecular simulation methods to predict the solubility of hydrogen and oxygen in PA6 and HDPE under high pressure. A comparative analysis of experimental results revealed that the solubility of hydrogen in PA6 and HDPE is lower than that of oxygen. Molecular simulations unveiled that oxygen exhibits more favorable energy interactions with PA6 and HDPE compared to hydrogen, and it is these interactions between gas molecules and polymers that predominantly govern gas solubility.

In addition to molecular dynamics, grand canonical Monte Carlo (GCMC) simulations are also commonly used to analyze the permeation mechanisms of molecules in polymers. Zheng et al. [172] employed GCMC simulations along with molecular dynamics methods to conduct a comprehensive study on hydrogen transport in PE pipelines under various factors such as temperature and pressure. The study revealed that the solubility, diffusion coefficient, and permeability of hydrogen in amorphous polyethylene are more significantly affected by temperature, and they all adhere to Arrhenius law. These findings were also corroborated by Zhang et al. [170].

**Figure 10:**
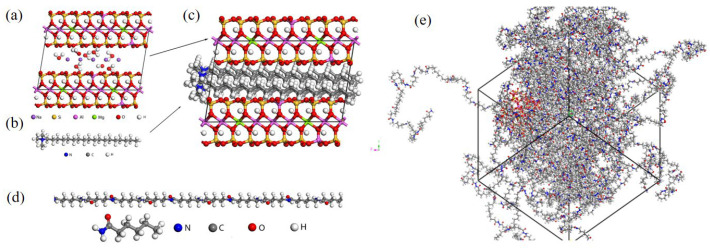
Molecular models: (**a**) original model of MMT, (**b**) octadecyl trimethylammonium chloride, and (**c**) montmorillonite model after surface modification; (**d**) one hundred repeating units of PA6 molecules; (**e**) composite model of PA6 single chains and MMT surface modification [173]. Copyright 2023, MDPI.

Materials Studio (MS) is the most favored molecular simulation software among researchers. Fang et al. [157] utilized MS molecular simulation software to construct molecular models of polyethylene and polyamide, simulating the diffusion and adsorption processes of hydrogen molecules in these two polymers at temperatures ranging from −10 to 80 °C. This revealed the permeation mechanisms of hydrogen in polyethylene and polyamide at the molecular scale, confirming the advantageous position of polyamide materials in resisting hydrogen permeation. As depicted in Figure 10, Wu et al. [173] employed MS software to construct models of organically modified montmorillonite (OMMT)-filled PA6, conducting in-depth explorations of helium adsorption and diffusion processes in the composite material system using molecular dynamics simulation methods. The study found that when the OMMT content was 5%, the composite material achieved the lowest permeability coefficient, and it reached the maximum energy barrier at a temperature of 55 °C and pressure of 41.6 MPa, exhibiting the best gas barrier properties. The continued pressure increase resulted in decreased gas barrier properties.

In summary, conducting in-depth research on the permeation mechanisms of Type IV hydrogen storage cylinders is of significant importance for advancing hydrogen energy technology, enhancing the performance and safety of hydrogen energy systems, achieving sustainable energy supply, and introducing new technologies.

### 3.3. Summary of Research Methods

In summary, the permeation behavior of hydrogen in Type IV hydrogen storage cylinder liners results from the synergistic influence of multiple factors. Researchers have employed a range of experimental and simulation methods—from macroscopic testing to microscopic mechanism analysis—to comprehensively investigate the gas permeation characteristics of plastic liners under various working conditions. Among experimental methods, the High-Pressure Hydrogen Permeation (HPHP) technique has become the mainstream standard due to its high accuracy and realistic simulation of actual service conditions. The Thermal Desorption Analysis (TDA) and pressure difference methods serve as complementary tools, showing advantages in rapid screening and low-pressure conditions. On the simulation side, molecular dynamics (MD) and grand canonical Monte Carlo (GCMC) simulations transcend the spatial and temporal limitations of experimental techniques, offering valuable insights into the microscopic diffusion paths and permeation mechanisms of polymer materials. To facilitate a comparison of the characteristics, advantages, and application scenarios of different research methods, the major experimental and simulation approaches for liner permeability are summarized in Table 3.

## 4. The Application and Challenges of Inner Liner Gas Permeability

### 4.1. The Safety of Hydrogen Storage Systems

The safety of hydrogen storage systems is of paramount importance, especially considering the high pressure, flammable, and explosive nature of hydrogen gas. Hence, stringent evaluations are imperative before the use of Type IV hydrogen storage cylinders. Material selection, structural design, and manufacturing processes must all adhere to rigorous standards and specifications [33,129]. The permeability of Type IV hydrogen storage cylinders must be fully considered to prevent hydrogen leakage. Regular inspection and testing of permeability can ensure the safe operation of hydrogen storage systems [148]. Additionally, leak detection and alarm systems should be installed in hydrogen storage systems to promptly detect and address any hydrogen leaks, minimizing potential safety risks. Ensuring that hydrogen systems are equipped with necessary emergency measures, including emergency shut-off valves, leak suppression systems, and adequate ventilation facilities, is essential for addressing emergencies and minimizing potential harm. The regular inspection, maintenance, and upkeep of hydrogen systems are crucial for ensuring their safety. This includes inspecting and maintaining components such as cylinders, pipelines, valves, and leak detection systems. All aspects of the design, manufacturing, installation, and operation of hydrogen systems must comply with applicable regulations and standards to ensure their safety and compliance [53,174,175,176].

**Figure 11:**
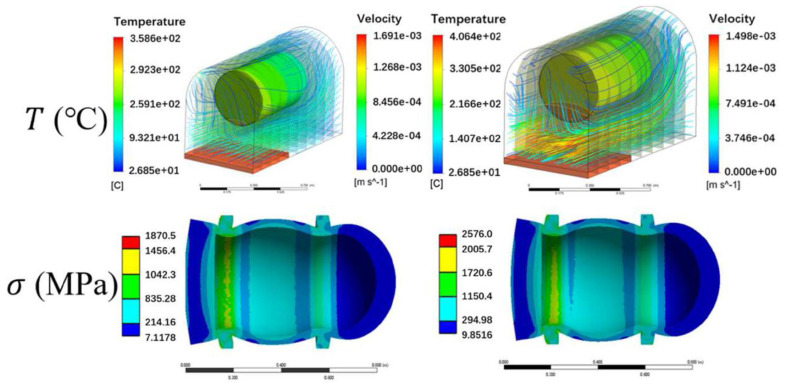
Temperature distribution across the entire ANSYS FLUENT model and corresponding stress on the cylinder wall [175]. Copyright 2024, Elsevier.

For instance, Huang et al. [177] established a mathematical model based on the R-K state equation for the safety requirements of rapid release of high-pressure hydrogen cylinders in emergency situations of hydrogen-powered vehicles. They analyzed the characteristics of the safety release valve circuit for hydrogen cylinders and compared them with the test results of fire tests, providing a theoretical basis for the design and analysis of safety release valve circuits for high-pressure hydrogen cylinders in hydrogen-powered vehicle applications. Kwan et al. [175] proposed a comprehensive 3D fire safety model for hydrogen cylinders, as depicted in Figure 11. By considering the volume heat source below the hydrogen cylinder and accounting for the complex influences of heat transfer, fluid dynamics, and solid walls of the installation space, they calculated temperature distribution on the cylinder wall with higher accuracy compared to existing models that oversimplify the fire source model. Similarly, scholars are also devoted to studying the safety operation of mobile hydrogen refueling stations. Li et al. [178] conducted simulation studies on the dispersion of hydrogen leaks and the minimum safe distance for explosion in mobile refueling stations, providing valuable guidance for the safe operation of such stations.

### 4.2. Manufacture and Application of Inner Liner

The molding process of the plastic liner in Type IV hydrogen storage tanks directly impacts the gas permeability. This is because different molding processes result in different process defects [129]. Although injection welding is a mature process, many well-known automotive companies worldwide choose to develop and use hydrogen storage tank liners manufactured using injection welding techniques [179]. However, high-polymer materials like PA6 tend to produce bubbles during the injection process. Moreover, the welded assembly of the liner parts after injection leads to stress concentration at the weld positions, shortening the liner’s lifespan. Additionally, uneven wall thickness can cause variations in permeability. Therefore, according to standards, the number of welds should not exceed two.

In response to the drawbacks brought by welding to the bottle body, a new molding technology called “roll forming” is being vigorously developed by enterprises [180,181,182]. The roll forming process does not involve weld seams in the molded liner, and the texture is relatively uniform. During the roll forming process, the liner can be directly connected to the metal BOSS. However, for roll forming to be successful, the raw material needs to have good fluidity after heating to ensure uniform wall thickness. If the raw material has high viscosity and poor flowability, it will be challenging to control the tightness of the connection between plastic and metal BOSS, as well as the uniformity of the wall thickness [183,184]. Furthermore, roll forming is considered a low-pressure forming technology, which makes it difficult to ensure the density of the formed parts and the material’s orientation for high-polymer materials. As a result, the gas barrier properties of the material may not be improved [129]. In the future, breaking through this technological barrier will require further exploration and research by more researchers.

Materials with higher orientation often possess better gas barrier properties. If the material undergoes biaxial stretching during the manufacturing process, its orientation can be enhanced [102]. Blow molding technology can improve the material’s gas barrier properties and impact resistance by enhancing its orientation [185,186].

**Figure 12:**
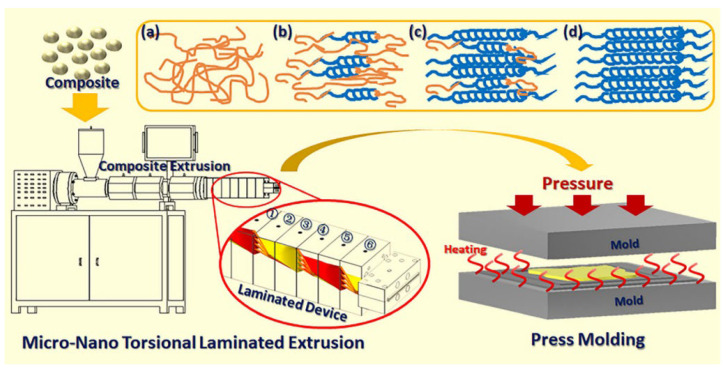
Schematic of the MNTLE and press-molding methods. (**a**) Random chain. (**b**) Interface orientation. (**c**) Thickness reduction of the single layer and enhanced center melt orientation. (**d**) Full orientation [115]. Copyright 2023, Wiley.

**Figure 13:**
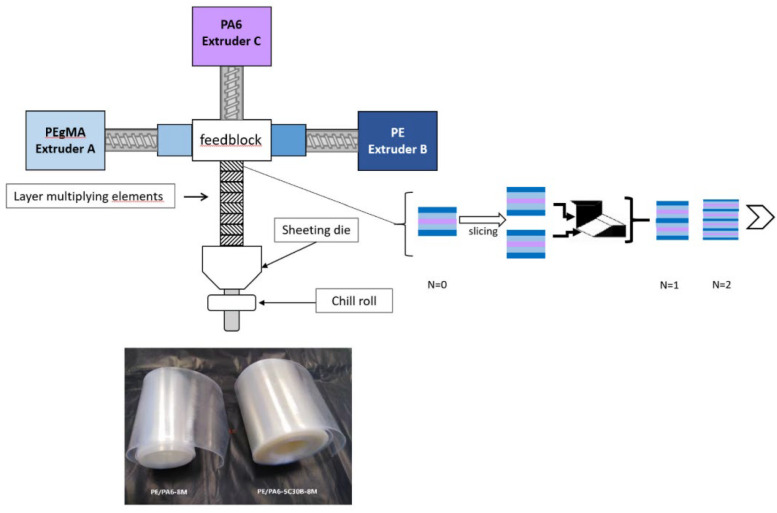
Schema of the forced assembly coextrusion line using three extruders for PE/PA6 multilayer films [63]. Copyright 2024, Elsevier.

To enhance the hydrogen barrier effect of the inner liner of Type IV hydrogen storage cylinders, research teams from various institutes are actively exploring new methods and technologies. For example, the MNTLE technology invented by Yang from Beijing University of Chemical Technology, as shown in Figure 12, utilizes three main steps: melt splitting, lamination, and stretching, to achieve multilayer lamination of polymer materials from single layers [115,129]. The materials processed by this technology have uniform texture and high orientation. The orderly arrangement of stretched molecular chains optimizes interstitial defects. During the processing of composite materials, it is possible to achieve uniform and staggered distribution of fillers, forming complex gas permeation paths, thus improving the gas barrier performance of polymer materials [62]. The Marais team developed a co-extrusion process with a layer multiplier element (LME), as shown in Figure 13, which enhances the edge orientation of crystals, thereby increasing the material’s barrier properties [63,121]. This technology provides new technical support and guarantees for the modification and development of materials with high-gas-barrier properties. Rajasekar et al. [128] prepared composite multilayer films containing graphene oxide (GO) using Layer-by-Layer (LBL) assembly technology, achieving a hydrogen permeability rate of 11.7 cc/m^2^ d atm, demonstrating high-hydrogen-barrier effects.

Clearly, the safety of hydrogen storage systems primarily relies on properly regulated operational methods, which can minimize safety risks and the potential for hydrogen leakage. Additionally, considerations should be given to the sustainability of hydrogen refueling station facilities and the hydrogen storage containers themselves, ensuring that advanced processing techniques and methods bring new possibilities for economic sustainability.

## 5. Limitations in Research on Inner Liner Gas Permeability

Although extensive research has been conducted on the gas permeability behavior of inner liner materials for Type IV hydrogen storage cylinders, several key limitations remain. Many studies are based on laboratory-scale experiments carried out under simplified conditions, typically involving pure hydrogen at constant pressure and temperature. Such conditions fail to represent the complex service environments, which include pressure cycling, temperature variation, and mechanical deformation.

Moreover, much of the existing work is focused on short-term testing and does not adequately address long-term aging effects, such as thermal oxidation, ultraviolet exposure, and chemical interactions with the composite overwrap. These factors can significantly influence the microstructure and gas barrier properties of liner materials. In addition, the relationship between gas permeability and microstructural characteristics—such as crystallinity, amorphous phase distribution, free volume, and chain mobility—has not been thoroughly explored. For semi-crystalline polymers like PA6, gas transport is strongly affected by the arrangement of crystalline and amorphous regions, yet quantitative data and predictive models are still limited.

To enable a more comprehensive understanding of liner materials under service conditions, future research should focus on integrating multi-factor environmental simulations, long-term aging evaluations, and structure–property correlation analysis related to gas transport mechanisms.

## 6. Future Perspectives

Looking ahead, Type IV hydrogen storage cylinders will continue evolving toward lightweight structures, higher operating pressures, and adaptability to extreme environments. Achieving these goals will depend heavily on improving the properties of liner materials.

The development of new polymer-based or hybrid materials with lower hydrogen permeability, enhanced thermal and mechanical stability, and better aging resistance will be key. Nanotechnology offers promising solutions in this regard. Through controlling the structural properties of nanomaterials, gas barrier performance can be significantly improved, thereby reducing hydrogen permeation. In parallel, advanced computational simulation and modeling tools are expected to play a more prominent role in elucidating gas transport mechanisms and guiding the rational design of liner materials. Such tools will also support the establishment of structure–property relationships and enable predictive material development through a feedback optimization loop.

In addition to performance improvements, future research should also address the scalability, cost-efficiency, and environmental sustainability of liner materials and their manufacturing processes. Emphasis will likely shift toward mass production techniques that ensure material uniformity and consistency, while minimizing cost and environmental impact.

Ultimately, progress in this field will rely on close collaboration between materials science, mechanical engineering, and computational modeling. This integration will open up new directions for hydrogen storage systems, supporting the expansion of hydrogen applications in fields such as transportation, aerospace, and deep-sea exploration.

## 7. Conclusions

The Type IV hydrogen storage cylinder is a critical component of modern hydrogen storage technology, and the gas permeability of its inner liner plays a vital role in ensuring the cylinder’s performance, safety, and long-term reliability. This review has summarized the current research progress on liner materials, including their structural characteristics, gas barrier performance, and behavior under various environmental conditions.

Despite advancements in materials development and characterization methods, several challenges remain unresolved. These include the relatively high gas permeation rates of existing materials, limited understanding of long-term aging mechanisms, and the trade-offs between permeability, mechanical integrity, and cost-effectiveness. These limitations hinder the large-scale deployment of hydrogen storage technologies, especially in safety-critical applications.

Therefore, further interdisciplinary research and technological innovation are essential to overcome these bottlenecks and promote the safe and efficient use of hydrogen energy.

## Figures and Tables

**Table 1:** Gas barrier and mechanical properties of polymeric materials.

Materials	Technical Means	Barrier Property	Mechanical Property	Ref.
Graphite/PE	Lamination		Tensile strength increased by 68.39% compared to pure PE.	[114]
PE/GF	Boundary constraint-free hot-pressing method	Helium gas permeability 3.92 × 10^−9^ Pa·m^3^/s.	Tensile strength 23.24 MPa, Young’s modulus 1.78 GPa.	[119]
PA6/α-ZrP	Injection or blow-molded	Helium permeability coefficient 0.8 × 10^−10^ cm_STP_ cm cm^−2^ s^−1^ cm_Hg_^−1^.		[120]
PE/PA6	A coextrusion process with layer multiplier elements (LME)	Combination of moisture resistance of PE and barrier properties of PA6.	Young’s modulus of 956 MPa, tensile strength of 37 MPa, elongation at break of 609%.	[121]
PA6/Cloisite	Coextrusion with layer multiplier elements (LMEs)	Permeability coefficient P: P_N2_ < P_O2_ < P_CO2_.	Fracture stress 80–85 MPa, Young’s modulus 0.9–1.0 GPa.	[63]
PE/PA6/Cloisite	A coextrusion process with layer multiplier elements (LME)	Improved oxygen barrier effect 58%.	Packing has an effect on Young’s modulus and elongation at break, but a much smaller effect on stress at break.	[63]
PA6	Injection molding according to GB/T 42610-2023 standards [122]	Hydrogen permeability coefficient 1.72 × 10^−14^ cm^3^·cm/cm^2^·s Pa.		[32]
PA11	Injection molding according to GB/T 42610-2023 standards [122]	Hydrogen permeability coefficient 1.87 × 10^−14^ cm^3^·cm/cm^2^·s Pa.		[32]
HDPE	Injection molding according to GB/T 42610-2023 standards [122]	Hydrogen permeability coefficient 5.88 × 10^−14^ cm^3^·cm/cm^2^·s Pa.		[32]
HDPE/MMT	MNTLE	Oxygen permeability coefficient 2.485 × 10^−14^ cm^3^·cm/cm^2^·s Pa.	Tensile Strength 35.42 MPa, Elongation at break 848.31%.	[62]
HDPE/HP030	Multi-layer extrusion technology	Oxygen barrier increased by 2 times, water vapor barrier increased by 5 times.		[123]
HDPE/PA6	Micro-layer coextrusion technology	Nitrogen permeability coefficient 2.89 × 10^−12^ cm^3^·cm/cm^2^·s Pa.		[124]
EVOH32		Oxygen permeability 6 cm^3^ mm/(m^2^ day atm).		[125]
EVOH/LDPE	Continuous multilayer coextrusion	OTR 0.60 ± 0.06 cm^3^/(m^2^ day).	Tensile strength 7.3 ± 0.3 MPa, elongation at break 202 ± 60%, Young’s modulus 181 ± 9 MPa.	[126]
HDPE	Hot-press method	Pressure 90 MPa Hydrogen permeability coefficient 4.95 × 10^16^ mol·m/(m^2^·s·Pa)		[127]
PDDA/SPVDF-GO	Layer-by-layer assembly (LBL)	Hydrogen transmission rate 11.7 cc/m^2^ d atm.	Tensile strength 366.2 MPa, Young’s modulus approx. 7 GPa, elongation at break approx. 220%.	[128]

**Table 2:** Summary of research on gas permeability of Type IV hydrogen storage cylinder liners.

Category	Content Summary
Research Theme	Comprehensive review of gas permeability in Type IV hydrogen cylinder liners, including materials, oxidation, pressure–temperature effects, and coating strategies.
Main Materials	PA6, PA11, PA12 (good mechanical/gas barrier); HDPE (easily processable but low barrier); and nanocomposites (e.g., PE + MMT, PE + GF, PE/PA multilayers) improved both strength and permeability.
Key Influencing Factors	Material selection, crystallinity, functional groups, multilayer structures, temperature–pressure conditions, thermal oxidation, and overwrap–liner interface.
Research Highlights	Nanofillers (e.g., GF, ZrP, MMT) significantly enhanced gas barrier properties, reducing hydrogen permeability by up to 98.6%; multilayer co-extrusion improved both tensile strength and gas resistance; mechanical performance was retained under low-pressure H_2_ conditions.
Limitations	Lack of studies simulating real service environments; insufficient understanding of multilayer interface failure (e.g., buckling, foaming); limited engineering-scale testing and standardization.
Application Areas	Primarily in fuel cell vehicles; potential for aerospace, deep-sea, and other extreme-environment hydrogen storage.
Future Outlook	Development of high-barrier polymer systems, functionalized nanocomposites, interface engineering, and multiphysics simulation; focus on scalable processing and material standardization.

**Table 3:** Summary of hydrogen permeation research methods.

Method Type	Representative Techniques	Advantages	Limitations	Application Scenarios	Ref.
**Experimental Methods**	High-Pressure Hydrogen Permeation (HPHP)	High accuracy; aligned with real service conditions; standardized	Time-consuming; requires specialized equipment	Standard qualification testing of liner materials	[32,122,148,159]
	Thermal Desorption Analysis (TDA)	Rapid hydrogen dissolution analysis; suitable for screening	Less accurate under high pressure; prone to distortion during decompression	Preliminary assessment of dissolved hydrogen content	[127]
	Pressure Difference Method	Suitable for low-permeability membranes; simple setup	Limited to low or moderate pressure; slower response under dynamic conditions	Membrane-level gas permeation testing	[62,124,125]
**Simulation Methods**	Molecular Dynamics (MD)	Microscopic insight; predicts free volume, diffusion behavior, and polymer–gas interactions	High computational cost; requires accurate force fields	Mechanism study, temperature/pressure effect evaluation	[168,169,170,171,173]
	Grand Canonical Monte Carlo (GCMC) + MD	Quantifies solubility and diffusion; effective under varying thermodynamic conditions	Less effective for non-equilibrium phenomena	Simulation of sorption and transport processes	[170,172]
	Materials Studio (MS) molecular modeling	Visual modeling of complex systems; useful for filler and nanocomposite studies	Requires expertise in simulation software and validation	Design of nanocomposite structures; prediction of barrier performance	[157,173]

## Data Availability

No new data were created or analyzed in this study. Data sharing is not applicable.
